# Editorial: Multimedia environmental pollution and food safety: New insights from integrated consumer nutrition and health risk management

**DOI:** 10.3389/fpubh.2023.1132998

**Published:** 2023-01-23

**Authors:** Fei Li, Chuanrong Zhang, Zhihua Xiao, Hao Hu

**Affiliations:** ^1^Research Center for Environment and Health, Zhongnan University of Economics and Law, Wuhan, Hubei, China; ^2^Department of Geography and Center for Environmental Sciences and Engineering, University of Connecticut, Storrs, CT, United States; ^3^College of Resources and Environment, Hunan Agricultural University, Changsha, China; ^4^College of Food Science and Technology, Huazhong Agricultural University, Wuhan, Hubei, China

**Keywords:** environmental pollution, consumer nutrition, health risk management, dietary strategies, food chemistry and safety

Environmental pollution and food safety are closely related critical issues faced by all populations over the world. Safe foods provide the necessary nutrients for human survival and health. Unfortunately, anthropogenic source-driven contaminants could be transported to foods (*via* crops, vegetables, fruits, and animals) from the contaminated multimedia environments including air, soil, surface water or groundwater, etc. This Research Topic aims to provide a platform for scholars to report their research progress on studying multimedia environmental pollutions and food safety around the world. Multidisciplinary research is urgently needed to explore a range of health and nutrition inquiries, from issues of the food supply chain to cooking practices and dietary safety management, not only in normal times but also as a function of the COVID-19 pandemic. The Research Topic has attracted wide attention and generated six multi-disciplinary articles in total.

In this Research Topic, a team from Cukurova University (Turkey) and North Carolina A & T State University (United States) conducted a literature review about the impact of COVID-19 pandemic on seafood safety and human health (Rathod et al.). Another team from University of Veterinary Medicine and Pharmacy (Slovakia) and Menoufifia University (Egypt) performed a health risk evaluation of organochlorine pesticide residues in edible tissue of seafood based on 120 random samples from local markets in Mansoura city, Egypt (Hussein et al.). Furthermore, the study by Chen et al. proposed a novel theoretical framework for food safety management problem using a case study of China's waste cooking oil (WCO). This paper bridged the research gap by systematically applying grounded theory to real criminal cases to explore the main potential influencing factors of WCO crime comprehensively. Zhang G. et al. from the Affiliated Zhongda Hospital of Southeast University (China) used data from the 2013–2014 National Health and Nutrition Examination Survey (NHANES) database to initially explore the relationship between urinary concentrations of Heterocyclic aromatic amines (HAAs) and the risk of kidney stones, which was validated by propensity score matching (PSM) analysis. For detecting botulinum toxin type A, which can cause serious food poisoning, in complex sample matrices. Zhang L. et al. from Anhui Medical University (China) introduced a rapid and sensitive detection tool using AlphaLISA, which was proved to a potentially rapid toxin detection method for foods. Finally, Guanqi et al. evaluated the role of organic food supply chain traceability in food safety and consumer wellbeing using a mediated-moderation investigation.

This Research Topic provides not only a timely reference source for academics, but also has practical implications for decision-makers concerned with environmental pollution and food safety. We thank the members of the Editorial Board and all authors and referees for their valuable contributions to this Research Topic. Of course, these publications would not be possible without the support from the Journal Office.

## Author contributions

FL wrote the draft of this manuscript. CZ, ZX, and HH reviewed and approved the final version. All authors contributed to the article and approved the submitted version.

